# Vitamin D Deficiency as a Contributing Factor to Chronic Rhinitis in Middle-Aged and Older Adults: An Epidemiological Study

**DOI:** 10.3390/nu16193385

**Published:** 2024-10-05

**Authors:** Sang Chul Park, Do-Yang Park

**Affiliations:** 1 Department of Otorhinolaryngology-Head and Neck Surgery, Kangnam Sacred Heart Hospital, Hallym University College of Medicine, Seoul 07441, Republic of Korea; newliebe@hanmail.net; 2 Department of Otolaryngology, Ajou University School of Medicine, Suwon 16499, Republic of Korea

**Keywords:** vitamin D deficiency, chronic rhinitis, aging, epidemiology

## Abstract

Background: Recent studies suggest a critical role for vitamin D in respiratory diseases, including asthma and allergic rhinitis. However, the relationship between vitamin D deficiency and chronic rhinitis, particularly in middle- and older-aged populations, remains underexplored. This study aimed to investigate the association between vitamin D deficiency and chronic rhinitis in middle- and older-aged adults while controlling for lifestyle and physical status factors. Methods: Data from 12,654 participants aged 40 years and older were analyzed from the fifth Korean National Health and Nutrition Examination Survey (2010–2012). The prevalence of chronic rhinitis and its association with serum vitamin D levels were assessed using multiple logistic regression models, adjusting for demographic, lifestyle, and physical characteristics. Results: The prevalence of chronic rhinitis was 21.1%. Participants with chronic rhinitis had a higher prevalence of vitamin D deficiency (69.9% vs. 65.2%) and lower mean vitamin D levels (17.73 ng/mL vs. 18.19 ng/mL) compared to those without chronic rhinitis. After adjusting for confounding factors, vitamin D deficiency remained significantly associated with an increased likelihood of chronic rhinitis (OR = 1.21, 95% CI, 1.082–1.348, *p* = 0.001). Conclusions: This study identifies a significant association between vitamin D deficiency and chronic rhinitis in middle- and older-aged adults, suggesting that maintaining adequate vitamin D levels may be important in managing chronic rhinitis.

## 1. Introduction

Chronic rhinitis is a persistent inflammatory condition of the nasal mucosa characterized by symptoms such as nasal congestion, rhinorrhea, sneezing, and itching that last for more than 12 weeks. It significantly impacts the quality of life, causing sleep disturbances, impaired daily functioning, and emotional distress. Rhinitis is generally divided into allergic rhinitis and non-allergic rhinitis [1]. Non-allergic rhinitis is a heterogeneous disease, independent of an IgE-mediated mechanism that includes atrophic rhinitis, vasomotor rhinitis, drug-induced rhinitis, gustatory rhinitis, and non-allergic rhinitis with eosinophilia (NARES) [2]. Allergic rhinitis seems to decrease with age, whereas non-allergic rhinitis increases with age, and the highest prevalence is seen in the elderly [3]. It is estimated that up to 200 million people worldwide suffer from non-allergic rhinitis [4].

Vitamin D regulates the metabolism of calcium and phosphorus and contributes to the maintenance of the musculoskeletal system. Moreover, most tissues and cells in the body have a vitamin D receptor; thus, various roles of vitamin D have been investigated [5]. Vitamin D has an immunomodulatory effect; affects innate and adaptive immunity as well as many immune cells, including B and T cells, dendritic cells, and monocytes/macrophages [6,7]. A recent meta-analysis study showed that vitamin D supplementation had protective effects against mortality and intensive care unit (ICU) admission in COVID-19 patients [8]. Furthermore, emerging experimental and clinical studies suggest an association between vitamin D and allergic rhinitis [9,10,11,12,13,14]. Although the results are inconsistent, several studies have suggested a slight tendency for serum vitamin D levels to be inversely associated with the risk of allergic rhinitis [15]. Nevertheless, a recent study has reported no significant association between vitamin D levels and allergic rhinitis, indicating the need for further investigation [16]. However, there is a significant gap in research regarding the relationship between vitamin D and chronic rhinitis, which is concerning given that chronic rhinitis lacks well-established treatment options. Understanding this relationship is crucial, as it could lead to new insights and potentially more effective management strategies for chronic rhinitis, particularly in the aging population where this condition is most prevalent. Middle-aged and older adults are more susceptible to chronic rhinitis due to age-related changes in the nasal mucosa, mucociliary clearance, and immune responses [3,17].

Therefore, in the present study, our primary objective was to examine the prevalence of vitamin D deficiency among middle-aged and older individuals with chronic rhinitis. Moreover, vitamin D has been found to be associated with several lifestyle and physical status factors in many studies. The lifestyle factors include smoking [18] and drinking history [19], regular exercise [20], and sleep duration [21]; physical status factors include obesity [22], hypertension [23], diabetes [24], and dyslipidemia [25]. Consequently, we aimed to investigate the relationship between lifestyle factors, physical health status, and vitamin D levels within this population. Furthermore, we sought to analyze the association between vitamin D deficiency and the occurrence of chronic rhinitis while carefully controlling for the potential confounding effects of lifestyle and physical health factors. By addressing these aspects, we hope to provide new insights into the role of vitamin D in managing chronic rhinitis, particularly in an aging demographic that is most affected by this condition.

## 2. Materials and Methods

### 2.1. Study Population and Data Collection

The Korea Centers for Disease Control and Prevention, in collaboration with the Korean Society of Otorhinolaryngology—Head and Neck Surgery and other relevant societies, have routinely assessed the medical history and clinical data of the Korean population through the Korean National Health and Nutrition Examination Survey (KNHANES). Established as a nationwide survey, the KNHANES has been conducted by the Korea Centers for Disease Control and Prevention since 1998 to evaluate the health and nutritional status of the general Korean population. The survey employs a multistage, stratified, cross-sectional sampling method, ensuring no overlap of subjects. Clinical examinations were carried out nationwide by teams of four medical professionals, including an otolaryngologist, using specially equipped mobile examination units. During a single visit, each participant completed questionnaires, provided samples, and underwent examinations. To ensure consistency across the survey, the KORL-HNS provided training to the survey teams, standardizing the examination procedures. Our study was conducted on KNHANES data obtained between 2010 and 2012 (n = 25,534). In total, 12,654 participants 40 years old or older were included in the final study population of the present study. Vitamin D metabolism undergoes significant alterations after middle age. These changes include decreased cutaneous synthesis, reduced sensitivity to active forms of vitamin D, and lower vitamin D receptor expression, which collectively impact immune function [26,27,28]. For this reason, we selected 40 as the cut-off age for this study. The survey protocol was approved by the institutional review board of the Korea Centers for Disease Control and Prevention (IRB Nos. 2010-02CON-21-C, 2011-02CON-06-C, and 201201EXP-01-2C). All study participants provided written informed consent as part of the KNHANES.

### 2.2. Assessment of Chronic Rhinitis

We included both participants who subjectively reported rhinitis symptoms and those who had findings of allergic rhinitis on endoscopic examination. In the questionnaire, rhinitis symptoms like runny nose, nasal congestion, sneezing, or itching were assessed. The items used in our study were ‘T_Q_CR’ (subjective rhinitis symptoms), ‘T_Nc_Bf 1~3’ (endoscopic examination items), and the generated variable ‘T_sAlgrn’ (Supplementary Table S1).

### 2.3. Measurement of Serum 25-Hydroxy Vitamin D Level

Blood samples were obtained via the antecubital vein in the morning after participants had fasted for at least 8 h. Immediately after collection, the samples were refrigerated and then transported under cold storage conditions to a central testing facility, where they were analyzed within 24 h. Serum 25-hydroxyvitamin D [25(OH)D] levels were quantified using a radioimmunoassay (25(OH)D^125^I RIA Kit; DiaSorin, Still Water, MN, USA) with a gamma-counter (1470 Wizard; PerkinElmer, Turku, Finland). The assay demonstrated inter-assay coefficients of variation of 11.7%, 10.5%, and 8.6% at concentrations of 21.47, 56.66, and 82.37 nmol/L, respectively. As part of the vitamin D Standardization Program, the KNHANES ensured that the measurement of 25(OH)D was standardized in accordance with the reference procedures developed by Ghent University and the Belgian National Institute of Standards and Technology [29]. Despite ongoing debate, a 25(OH)D concentration below 20 ng/mL is widely recognized as indicative of vitamin D deficiency [30,31,32].

### 2.4. Statistical Analysis

We employed the SAS software (version 9.4; SAS Institute, Cary, NC, USA) to account for the complex sampling structure and survey weights derived from the KNHANES dataset. This method took into consideration unequal selection probabilities, oversampling, and non-responses. Descriptive statistics for participants’ characteristics are presented as means with standard errors for continuous variables, and frequencies with percentages for categorical variables. To investigate the association between vitamin D levels and chronic rhinitis, we conducted multiple logistic regression analyses using the PROC SURVEYLOGISTIC function in SAS.

We developed five models, each controlling for different sets of covariates to isolate the specific contribution of Vitamin D deficiency to chronic rhinitis risk. The crude model (Model 1) was unadjusted, serving as the baseline comparison. In Model 2, adjustments were made for age and sex, considering that these demographic factors could influence both Vitamin D levels and chronic rhinitis prevalence. In Model 3, we introduced lifestyle factors, including smoking status, alcohol consumption, regular physical activity, and sleep duration. These variables were included to account for behavioral patterns that could confound the relationship between Vitamin D deficiency and chronic rhinitis. Model 4 further adjusted for physical health conditions such as obesity, hypertension, diabetes, hyperlipidemia, hypercholesterolemia, and hypertriglyceridemia, recognizing that these comorbidities might affect both Vitamin D metabolism and susceptibility to chronic rhinitis. Finally, Model 5 incorporated all covariates from the previous models, thus fully adjusting for demographic, lifestyle, and physical health factors. This stepwise modeling approach allowed us to examine how the relationship between Vitamin D deficiency and chronic rhinitis changed as we controlled for potential confounders in each model. All statistical tests were two-tailed, and statistical significance was determined at *p* < 0.05.

## 3. Results

### 3.1. Baseline Demographic and Lifestyle Factors According to Chronic Rhinitis

**Figure 1:**
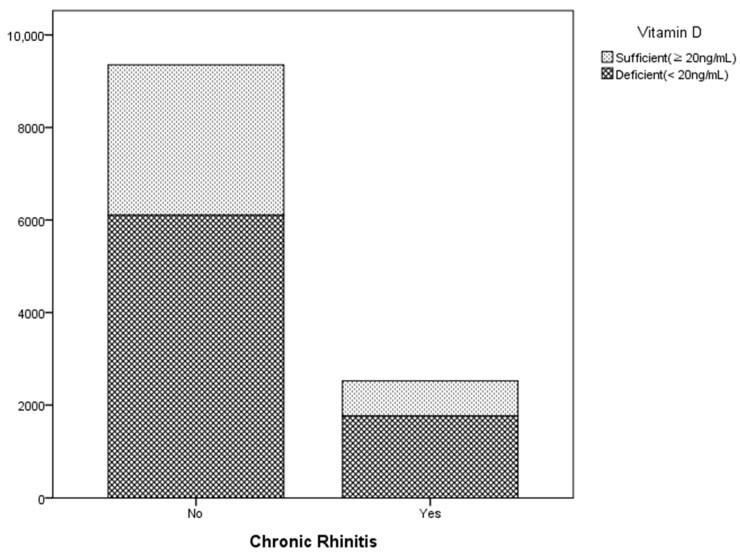
The relationship between chronic rhinitis and the number of individuals with sufficient or deficient vitamin D levels.

Among the 12,654 participants included in this study, 2668 (21.0%) reported having chronic rhinitis, while 9986 (78.9%) did not (Table 1). There was a significant difference in age distribution between the two groups (*p* < 0.001). Participants with chronic rhinitis were more prevalent in the 40–49-year age group (29.5%), with a decreasing prevalence observed in older age groups. Additionally, hypertension was significantly more common in individuals without chronic rhinitis (33.2%) compared to those with chronic rhinitis (30.3%) (*p* = 0.003). Notably, the vitamin D levels were significantly associated with chronic rhinitis (*p* < 0.001) (Figure 1); participants with chronic rhinitis had a higher prevalence of vitamin D deficiency (<20 ng/mL), at 69.9%, compared to 65.2% in those without chronic rhinitis. In contrast, other factors did not show statistically significant associations with chronic rhinitis.

### 3.2. Demographic and Lifestyle Factors on Vitamin D Status and Its Association with Chronic Rhinitis

A significant relationship was found between vitamin D levels and the presence of chronic rhinitis. Participants with chronic rhinitis had lower average vitamin D levels (17.73 ng/mL) compared to those without chronic rhinitis (18.19 ng/mL), and the prevalence of chronic rhinitis was higher in the vitamin D-deficient group (22.4%) than in the vitamin D-sufficient group (18.9%) (*p* < 0.001) (Table 2). In addition, age distribution showed significant differences (*p* < 0.001), with vitamin D deficiency increasing as participants’ age decreased. Furthermore, males were more likely to have sufficient vitamin D levels than females (*p* < 0.001). Obesity status (*p* = 0.037), hypertension (*p* = 0.005), hypertriglyceridemia (*p* = 0.002), smoking status (*p* = 0.002), alcohol consumption (*p* < 0.001), and regular exercise at both hard (*p* = 0.001) and moderate (*p* = 0.001) levels were also significantly associated with vitamin D levels. In contrast, diabetes mellitus, hyperlipidemia, hypercholesterolemia, and sleep duration did not show statistically significant differences between the groups.

### 3.3. Multiple Logistic Regression Analyses of Vitamin D Status Deficiency and Chronic Rhinitis

The relationship between chronic rhinitis and vitamin D deficiency was analyzed across five models, each controlling for various demographic, lifestyle, and physical health factors. Even after adjusting for confounders, individuals with vitamin D deficiency had an odds ratio of 1.21 to 1.25 (95% CI) for having chronic rhinitis compared with those not having chronic rhinitis (Table 3). The final model (Model 5), which adjusted for age, sex, lifestyle factors (including smoking, alcohol consumption, regular exercise, and sleep duration), and physical health status (including obesity, hypertension, diabetes, hyperlipidemia, hypercholesterolemia, and hypertriglyceridemia), demonstrated a statistically significant association. In this model, individuals with chronic rhinitis had an odds ratio (OR) of 1.21 (95% CI, 1.082–1.348, *p* = 0.001) for vitamin D deficiency compared to those without chronic rhinitis. This suggests that the likelihood of vitamin D deficiency is approximately 21% higher in individuals with chronic rhinitis compared to those without chronic rhinitis.

## 4. Discussion

In this study, we found a significant relationship between chronic rhinitis and vitamin D deficiency in middle- and old-aged populations. The prevalence of vitamin D deficiency was 69.9% among those with chronic rhinitis, higher than the 65.2% observed in participants without chronic rhinitis. Even after adjusting for confounders, subjects with low vitamin D levels were more likely to have chronic rhinitis. Participants with chronic rhinitis had a significantly higher likelihood of vitamin D deficiency, with an odds ratio indicating approximately 21% to 25% higher risk.

This finding aligns with existing research that underscores the role of vitamin D in respiratory diseases such as chronic rhinosinusitis, asthma, and chronic obstructive pulmonary disease [33,34]. Several studies have examined the connection between vitamin D and allergic rhinitis. Although some studies found no significant link between serum 25-hydroxyvitamin D levels and allergic rhinitis [9,16], other studies have suggested a tendency for vitamin D levels to be inversely associated with the risk of allergic rhinitis. Systematic reviews have supported the idea that vitamin D might help prevent allergic diseases, including allergic rhinitis, with evidence showing an inverse relationship between vitamin D levels and allergic rhinitis, particularly in pediatric populations [11,12]. One study has also shown that vitamin D supplementation can improve allergic rhinitis symptoms, indicating a potential therapeutic role [10]. Lower vitamin D levels have been associated with a higher prevalence of allergic rhinitis, with additional studies highlighting vitamin D’s potential in modulating immune responses, which could be relevant for managing allergic conditions [13,14,15]. The overall prevalence of chronic rhinitis in the general population was 40%, with 65% of those cases attributed to non-allergic rhinitis, 28% to allergic rhinitis, and 7% with an undetermined allergy status [2].

The potential mechanisms underlying the association between vitamin D deficiency and chronic rhinitis are rooted in vitamin D’s role in the immune system. Vitamin D enhances the antimicrobial response of macrophages and dendritic cells while also promoting the differentiation of Tregs, which help maintain immune tolerance and prevent excessive inflammatory responses [6,15,34]. Studies have shown that vitamin D can inhibit the production of pro-inflammatory cytokines such as IL-17, which are involved in the pathogenesis of chronic rhinitis and other inflammatory diseases [34]. Furthermore, vitamin D promotes the repair and proliferation of respiratory epithelial cells [35]. In the context of chronic rhinitis, vitamin D deficiency may lead to an imbalance in these immune processes, resulting in chronic inflammation of the nasal mucosa.

Additionally, the present study suggests that vitamin D deficiency may be associated with chronic rhinitis, particularly non-allergic rhinitis, through its impact on the autonomic nervous system (ANS). Recent studies have shown that vitamin D deficiency can negatively affect ANS function, leading to impaired cardiovascular autonomic regulation. For instance, Canpolat et al. demonstrated that individuals with vitamin D deficiency exhibit significant impairments in the heart rate recovery index (HRRI) and heart rate variability (HRV), both of which are key indicators of autonomic function [36]. Similarly, Dimova et al. highlighted the role of vitamin D in regulating neurotransmitter biosynthesis in the central nervous system, which is critical for maintaining cardiovascular autonomic function [37]. Given the role of the ANS in regulating inflammatory responses and mucosal function in the nasal passages, it is plausible that disturbances in ANS function due to vitamin D deficiency could contribute to the pathophysiology of chronic rhinitis. Non-allergic rhinitis, in particular, may be influenced by these autonomic dysfunctions, as it is characterized by symptoms that are often exacerbated by autonomic imbalances, such as vasomotor instability. Therefore, vitamin D deficiency may exacerbate or contribute to the development of non-allergic rhinitis by disrupting the normal autonomic regulation of the nasal mucosa.

Additionally, vitamin D’s ability to reduce oxidative stress and matrix metalloproteinase activity may help protect against tissue damage in the nasal mucosa, further supporting its protective role in chronic rhinitis [38,39].

In this study, the prevalence of vit D deficiency was high, with rates comparable to another study in Korea reporting 59.7% in men and 86.5% in women, with vitamin D deficiency defined as 25(OH)D < 20 ng/mL [40]. This is likely due to factors such as avoiding direct exposure to sunlight, higher skin pigmentation, and low vitamin D supplementation compared with Western populations.

This study has some limitations. First, there may be some ambiguity in the diagnostic criteria, as observed in various epidemiological studies. Our definition of chronic rhinitis includes both allergic and non-allergic rhinitis. The presence of chronic rhinitis was determined based on a questionnaire survey and endoscopic findings indicative of allergic rhinitis. However, using a questionnaire survey may introduce potential biases due to reliance on self-reported data. Second, as a cross-sectional study, we lacked information on the timing of rhinitis symptoms in participants. Third, longitudinal studies are necessary to confirm the causality between vitamin D deficiency and chronic rhinitis.

Nevertheless, this study has several strengths related to the investigation of chronic rhinitis and vitamin D. It was conducted with a large sample size and utilized a nationally representative dataset, which enhances the generalizability of the results in the field of rhinology. Additionally, this study included comprehensive adjustments for variables such as age, sex, lifestyle factors, and physical status.

These findings suggest that maintaining adequate vitamin D levels could be a key factor in managing chronic rhinitis and potentially other related respiratory conditions. Although these results do not imply a specific etiological or therapeutic relationship, vitamin D may represent an inexpensive and cost-effective option for controlling chronic rhinitis, either alone or as a synergistic agent with traditional therapies. Further research is needed to elucidate the precise pathways through which vitamin D influences these processes and to determine the optimal levels of vitamin D for preventing and treating chronic rhinitis. We suggest that future prospective studies should investigate the potential preventive effects of vitamin D supplementation in reducing the risk of chronic rhinitis in individuals with low vitamin D levels, particularly in smokers and alcohol consumers.

## 5. Conclusions

In summary, this study highlights a significant association between vitamin D deficiency and the prevalence of chronic rhinitis. These findings suggest that individuals with chronic rhinitis are at a greater risk of vitamin D insufficiency, which could play a crucial role in the pathophysiology of the condition. However, further prospective studies are needed to confirm this association. By focusing on a relatively underexplored aspect of chronic rhinitis, this research provides important insights that could inform future clinical practices. Further research is needed to clarify the mechanisms by which vitamin D impacts chronic rhinitis, to better inform targeted interventions.

## Figures and Tables

**Table 1:** Baseline characteristics according to chronic rhinitis.

Variable		Chronic Rhinitis		
		Yes n (%)	No n (%)	*p*-Value
N		2668 (21.1%)	9986 (78.9%)	
Age (year)	40–49	788 (29.5%)	2386 (23.9%)	<0.001
	50–59	767 (28.7%)	2660 (26.6%)	
	60–69	583 (21.9%)	2502 (25.1%)	
	70–79	445 (16.7%)	1975 (19.8%)	
	80–89	80 (3.0%)	454 (4.5%)	
	≥90	5 (0.2%)	9 (0.1%)	
Sex	Male	1187(44.5%)	4263 (42.7%)	0.095
	Female in menopause	1481 (55.5%)	5723 (57.3%)	
Obesity	Underweight	76 (2.9%)	294 (2.9%)	0.011
	Normal	1734 (65.1%)	6176 (62.0%)	
	Overweight	853 (32.0%)	3497 (35.1%)	
Hypertension	No	1860 (69.7%)	6665 (66.7%)	0.003
	Yes	808 (30.3%)	3321 (33.2%)	
Diabetes	No	2383 (89.3%)	8794 (88.1%)	0.073
	Yes	285 (10.7%)	1192 (11.9%)	
Hyperlipidemia	No	2281 (85.5%)	8622 (86.4%)	0.269
	Yes	386 (14.5%)	1362 (13.6%)	
Hypercholesterolemia	No	1982 (81.9%)	7222 (80.8%)	0.206
	Yes	438 (18.1%)	1720 (19.2%)	
Hypertriglyceridemia	No	1767 (83.7%)	6495 (83.0%)	0.457
	Yes	345 (16.3%)	1332 (17.0%)	
Smoking status	No	2124 (82.0%)	7967 (82.5%)	0.606
	Yes	465 (18.0%)	1693 (17.5%)	
Alcohol drinking	No	1333 (51.6%)	5137 (53.4%)	0.121
	Yes	1248 (48.4%)	4490 (46.6%)	
Regular exercise (hard)	No	2252 (87.0%)	8426 (87.4%)	0.649
	Yes	336 (13.0%)	859 (12.6%)	
Regular exercise (moderate)	No	2372 (91.8%)	8790 (91.1%)	0.264
	Yes	212 (8.2%)	1220 (8.9%)	
Sleep duration (hour)	<6	508 (20.2%)	1823 (19.4%)	0.252
	6 to 9	1914 (75.9%)	7126 (75.9%)	
	≥10	99 (3.9%)	435 (4.6%)	
Vitamin D level (ng/mL)	<20	1764 (69.9%)	6103 (65.2%)	<0.001
	≥20	758 (30.1%)	3252 (34.8%)	

**Table 2:** Vitamin D levels according to participant characteristics.

Variable		Vit. D (ng/mL)	Vit. D (ng/mL) <20 ng/mL	Vit. D (ng/mL) >20 ng/mL	*p*-Value
		Geometric Mean	%	%	
Age (year)	40–49	16.75	29.9	18.7	<0.001
	50–59	18.25	28.3	27.5	
	60–69	19.03	22.3	29.0	
	70–79	18.91	16.6	21.2	
	80–89	18.99	2.8	3.5	
	≥90	17.35	0.1	0.1	
Sex	Male	19.36	38.8	53.6	<0.001
	Female	17.28	61.2	46.4	
Obesity	Underweight	18.32	2.5	2.9	0.037
	Normal	18.26	62.1	63.9	
	Overweight	18.07	35.3	33.1	
Hypertension	No	18.08	69.2	66.7	0.005
	Yes	18.43	30.8	33.3	
Diabetes	No	18.17	89.0	88.3	0.246
	Yes	18.33	11.0	11.7	
Hyperlipidemia	No	18.15	86.7	85.7	0.161
	Yes	18.44	13.3	14.3	
Hypercholesterolemia	No	18.25	81.0	81.2	0.773
	Yes	18.13	19.0	18.8	
Hypertriglyceridemia	No	18.31	82.3	84.7	0.002
	Yes	17.68	17.7	15.3	
Smoking status	Non-smoker	18.11	82.8	80.4	0.002
	Smoker	18.71	17.2	19.6	
Alcohol drinking	No	17.76	53.6	48.5	<0.001
	Yes	18.71	46.4	51.5	
Regular exercise (hard)	No	18.11	87.6	85.4	0.001
	Yes	18.87	12.4	14.6	
Regular exercise (moderate)	No	18.11	91.7	89.9	0.001
	Yes	19.27	8.3	10.1	
Sleep duration (hour)	<6	18.02	19.5	18.5	0.13
	6 to 9	18.20	76.5	76.9	
	≥10	18.71	4.0	4.7	
Chronic rhinitis	No	18.19	77.6	81.1	<0.001
	Yes	17.73	22.4	18.9	

**Table 3:** Association between chronic rhinitis and vitamin D deficiency.

Parameter		Model 1			Model 2			Model 3			Model 4			Model 5		
		OR	95% CI	*p*-Value	OR	95% CI	*p*-Value	OR	95% CI	*p*-Value	OR	95% CI	*p*-Value	OR	95% CI	*p*-Value
Chronic rhinitis	Yes	1.24	1.127–1.364	<0.001	1.22	1.105–1.342	<0.001	1.25	1.132–1.379	<0.001	1.24	1.115–1.377	<0.001	1.21	1.082–1.348	0.001
	No	1 (Ref)			1 (Ref)			1 (Ref)			1 (Ref)			1 (Ref)		

Model 1: crude model; Model 2: adjusted for age and sex; Model 3: adjusted for lifestyle factors (smoking status, alcohol-drinking status, regular exercise, and sleep duration); Model 4: adjusted for physical status (obesity, prevalent hypertension, prevalent diabetes, prevalent hyperlipidemia, prevalent hypercholesterolemia, and prevalent hypertriglyceridemia); Model 5: adjusted for age, sex, lifestyle factors, and physical status. OR = 1 for the ‘No’ group indicates the reference group, against which the ‘Yes’ group is compared. ‘Ref’ signifies that no confidence interval is provided for the reference group.

## Data Availability

All relevant data are included in the paper and its Supplementary Materials.

## Supplementary Materials

The following supporting information can be downloaded at: https://www.mdpi.com/article/10.3390/nu16193385/s1, Table S1: Questionnaire for chronic rhinitis; Supporting Excel File: Analysis results.
