# Supplementary material for: Vitamin D Deficiency as a Contributing Factor to Chronic Rhinitis in Middle-Aged and Older Adults: An Epidemiological Study
Source: Nutrients. 2024 Oct 5;16(19):3385. doi: 10.3390/nu16193385 (PMC11478465; doi:10.3390/nu16193385)

**Supplementary Table S1.** Questionnaire for chronic rhinitis in the Korean National Health and Nutrition Examination Survey (English Translation and Korean Original Version)

| Variable Type | Variable Name | Variable Description | Content |
| --- | --- | --- | --- |
| N (1) | T_Q_CR | Presence of Rhinitis Symptoms  "Have you experienced symptoms of rhinitis, such as a runny nose, nasal congestion, sneezing, or itching, without a cold (fever, sore throat) in the past year?" | 1. Yes  2. No  8. Not applicable (Only for those aged 6 and under)  9. No response |
| N (1) | T_Q_CR1 | Rhinitis Symptoms by Season | 1. Only in a specific season  2. Appears throughout the year  8. Not applicable (Only for those aged 6 and under)  9. No response |
| N (1) | T_Q_CR2 | Duration of Rhinitis Symptoms in the Week | 1. Only 1-4 days  2. 5days or more  8. Not applicable (Only for those aged 6 and under)  9. No response |
| N (1) | T_Q_CR3 | Duration of Rhinitis Symptoms in the Year | 1. Less than 1 year  2. 1 year or more  8. Not applicable (Only for those aged 6 and under)  9. No response |
| N (1) | T_Q_CR4 | Impact of Rhinitis Symptoms on Study/Work/Sleep | 1. Yes  2. No  8. Not applicable (Only for those aged 6 and under)  9. No response |
| N (1) | T_Nc_bf1 | Endoscopic Examination  : Pale Mucosa | 1. No  2. Yes  8. Not applicable (Only for those aged 6 and under)  9. No response |
| N (1) | T_Nc_bf2 | Endoscopic Examination  : Watery Rhinorrhea | 1. No  2. Yes  8. Not applicable (Only for those aged 6 and under)  9. No response |
| N (1) | T_Nc_bf3 | Endoscopic Examination  : Mucoid or Purulent Rhinorrhea | 1. No  2. Yes  8. Not applicable (Only for those aged 6 and under)  9. No response |
| N (1)* | T_sAlgrn | Self-Reported Presence of Allergic Rhinitis | 1. No  2. Yes |

* Generated Variables


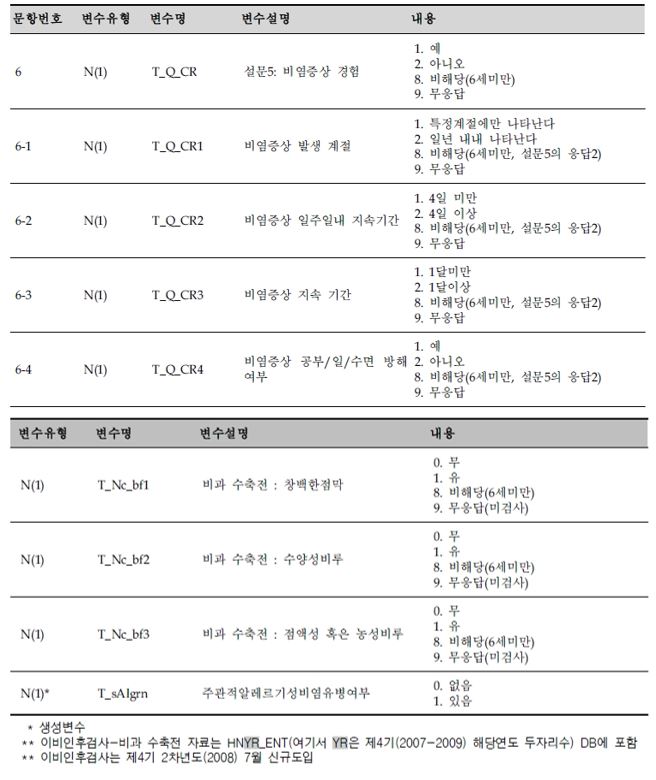

Supplement: Supplementary file 1 [file nutrients-16-03385-s001.zip › VitD Rhinitis Revision - Supplementary Table.docx]
